# Optimizing hemostasis to prevent bleeding and reduce transfusion requirements in neonatal and infant cardiac surgery with cardiopulmonary bypass: a systematic review and network meta-analysis

**DOI:** 10.3389/fped.2026.1823450

**Published:** 2026-06-10

**Authors:** Xiali Lu, Jiejuan Zhang, Jing Pan

**Affiliations:** 1 Department of Critical Care Medicine, West China Hospital/West China School of Nursing, Sichuan University, Chengdu, China; 2 Department of Respiratory and Critical Care Medicine, West China Hospital, Sichuan University, Chengdu, China

**Keywords:** cardiac surgery, cardiopulmonary bypass, hemostasis, infant, neonatal, network meta-analysis

## Abstract

**Background:**

Effective hemostatic management remained a critical challenge in neonatal and infant cardiac surgery with cardiopulmonary bypass (CPB), and a systematic review and network meta-analysis (NMA) had not been conducted to evaluate optimal strategy selection for this vulnerable population.

**Methods:**

We systematically searched PubMed, Web of Science, Cochrane Library, and Embase from inception to December 3, 2025. We use bivariate analysis and NMA with random effects. We use the surface beneath the cumulative ranking curve (SUCRA) to display the order of interventions.

**Results:**

Of the 8,280 records screened, 27 studies involving 4,556 patients were included. Aprotinin (APR) was identified as the optimal hemostatic strategy for reducing 24-hour blood loss (SUCRA=99.15%), platelet (PLT) (SUCRA=82.19%) and fresh frozen plasma (FFP) (SUCRA=84.82%) transfusion requirements, re-sternotomy for hemostasis (SUCRA=72.85%), and risks of thrombosis (SUCRA=79.13%) and renal dysfunction (SUCRA=72.61%). ε-aminocaproic acid (EACA) was found to be the most effective strategy for reducing red blood cell (RBC) transfusion needs (SUCRA=69.04%). Tranexamic acid (TXA) emerged as the optimal intervention for reducing mortality (SUCRA=84.77%).

**Conclusion:**

Antifibrinolytic agents may demonstrated significant hemostatic efficacy and a generally acceptable safety profile in neonatal and infant cardiac surgery with CPB. Among these agents, while APR showed the highest efficacy, its use is constrained by regulatory restrictions and evidence limitations; therefore, it should be used with caution. TXA remains a practical first-line alternative. In the available network, blood component therapies did not emerge as consistently superior strategies compared with other hemostatic interventions. For 24-hour blood loss, APR was associated with lower blood loss than FFP, FC, and PCC. However, because the relevant comparisons were largely against active comparators rather than placebo or no hemostatic intervention, the efficacy of blood component therapies relative to no blood component therapy remains uncertain. Future large-scale RCTs are needed to further validate these findings.

## 1 Introduction

Between 1970 and 2017, the global prevalence of unrepaired congenital heart disease among school-aged children (4–18 years) was 3.809 per 1,000 (1). These anomalies included atrial septal defect, ventricular septal defect, transposition of the great arteries, tetralogy of Fallot, hypoplastic left heart syndrome, and atrioventricular septal defect (2, 3). Most of these procedures required cardiopulmonary bypass (CPB). A number of studies have associated CPB with an increased risk of bleeding, exacerbated by factors such as coagulopathy, hypothermia, hemodilution, systemic inflammatory response, and therapeutic anticoagulation (4–6). This risk was particularly critical for neonates and infants undergoing CPB. Their immature coagulation systems and platelet function, combined with lower levels of most hemostatic proteins compared to older children, heightened their vulnerability (7). Furthermore, CPB-associated bleeding contributed to hemodynamic instability, delayed sternal closure, and increased transfusion requirements (8). Consequently, given these concerns, many recent studies have advocated for prophylactic hemostatic interventions, including antifibrinolytic agents and blood component therapies, as essential for reducing bleeding risk in neonates and infants undergoing CPB cardiac surgery (9, 10).

Hemostatic agents and blood component therapies play fundamental roles in preventing and controlling bleeding following cardiac surgery with CPB in neonates and infants. Clinicians select appropriate hemostatic strategies based on clinical observations, consideration of multiple factors, and evaluation of laboratory or point-of-care data. Commonly used hemostatic agents included tranexamic acid (TXA), ε-aminocaproic acid (EACA), and aprotinin (APR). TXA and EACA were demonstrated to reduce blood loss and transfusion requirements in various surgical settings without being associated with an increase in adverse events (11). APR, a potent broad-spectrum protease inhibitor, is effective in reducing perioperative bleeding and allogeneic blood transfusion requirements in high-risk cardiac surgery (12). However, existing data indicated that adults receiving APR faced an elevated risk of thrombosis, acute kidney injury, and mortality post-cardiac surgery (13–15). Although APR re-entered the European market in February 2016 (16), it remains unavailable in some countries or is approved only under limited indications, such as myocardial revascularization (particularly high-risk isolated coronary artery bypass grafting surgery) and in cardiac surgery patients at high risk for excessive bleeding (17, 18). Furthermore, new guidelines recommend enhanced monitoring of activated clotting time (ACT) and the establishment of a registry for aprotinin use (13). In recent years, driven by the pursuit of precision therapy and concerns over the severe complications associated with allogeneic empirical transfusion (19), blood component therapies gained increasing attention. Options applicable to neonatal and infant CPB cardiac surgery commonly included fresh frozen plasma (FFP), fibrinogen concentrate (FC), prothrombin complex concentrates (PCC), and recombinant activated factor VII (rFVIIa). In a methodological quality assessment of nine blood management guidelines for patients undergoing CPB, Huang et al. found that rFVIIa and desmopressin (DDAVP) were not recommended for use following pediatric cardiac surgery, while PCC could be considered within the context of clinical trials (20).

Recent systematic reviews and meta-analyses evaluated the efficacy and safety of TXA, EACA, APR, and FFP in pediatric cardiac surgery patients. Zou et al., in their analysis of 16 studies, found that TXA significantly increased postoperative platelet counts and reduced postoperative blood loss in pediatric patients undergoing cardiac surgery with CPB (21). Although multiple studies have confirmed that APR effectively controls postoperative bleeding (22, 23), the evidence regarding its impact on patient prognosis and adverse effects (such as thrombosis and renal dysfunction) remains inconclusive. The above studies found no significant differences compared with control groups, meaning that outcomes such as prognosis and safety remain a key focus of ongoing research. Schertz et al., who included 68 studies, reported that antifibrinolytic agents (TXA, EACA, APR) effectively reduced blood loss and blood product requirements in children undergoing CPB cardiac surgery (9). Kesumarini et al., in an analysis of 5 RCTs, found that using FFP in the cardiopulmonary bypass prime did not reduce postoperative blood loss in pediatric cardiac surgery patients overall; however, infants aged <7 months and weighing <6 kg were identified as a potential subgroup that might benefit (24). Given the considerable heterogeneity across studies in terms of interventions, a network meta-analysis was warranted to integrate both direct and indirect evidence, thereby addressing the limitation of conventional pairwise meta-analyses which can only compare two interventions at a time.

Therefore, this study aimed to conduct a systematic review and network meta-analysis to evaluate the effects of different hemostatic strategies on bleeding and transfusion requirements in neonates and infants undergoing cardiac surgery with CPB, as well as their impact on patient prognosis and safety. The findings were intended to provide an evidence-based foundation for clinical decision-making and the development of future guidelines.

## 2 Methods

This study was conducted in strict accordance with the principles outlined in the PRISMA guidelines (25). See Supplementary Table S1. The study protocol was prospectively registered with the International Prospective Register of Systematic Reviews (PROSPERO), registration number CRD420261279528 (https://www.crd.york.ac.uk/PROSPERO/).

### 2.1 Data sources and searches

This study systematically searched four electronic databases: PubMed, Embase, the Cochrane Library, and Web of Science. The search encompassed all available records from the inception of each database until December 3, 2025. Search terms included “Newborn,” “Infant,” “Cardiac Surgical Procedures,” “Thoracic Surgical Procedures,” “Cardiopulmonary Bypass,” and “Hemostasis.” See Supplementary Supplementary Table S2 for the complete search strategy. Truncation symbols (e.g., “*”) were used to maximize search sensitivity. Additionally, we manually screened the reference lists of previously published systematic reviews and meta-analyses to identify any potentially relevant studies that might have been missed by the electronic search.

### 2.2 Inclusion and exclusion criteria

Studies were considered eligible if they met the following criteria: (1) participants were neonates and infants undergoing cardiac surgery with CPB(average age ≤ 12 months); (2) interventions included antifibrinolytic agents (TXA, EACA, APR) or blood component therapies [FFP, FC, PCC, rFVIIa, a4FPCC (4-factor prothrombin complex concentrate)]; (3) study designs were randomized controlled trials (RCTs) or cohort studies; (4) at least one relevant outcome related to hemostasis, transfusion requirements, prognosis, or safety was reported, including 24-hour blood loss, requirements for red blood cell (RBC), platelet (PLT), or FFP transfusion, mortality, re-sternotomy for hemostasis, thrombosis, or renal dysfunction. The primary outcomes were 24-hour blood loss and transfusion requirements. Transfusion requirements were defined as the total volume (in mL or mL/kg) or the number of units of red blood cells, platelets, or fresh frozen plasma administered within 24 h postoperatively.

The exclusion criteria were as follows: studies based on animal experiments, systematic reviews, letters, or case reports; duplicate publications; studies not published in English; studies lacking baseline or original data for extraction; and studies for which the full text was unavailable or only an abstract was reported.

### 2.3 Study selection and data extraction

All literature was managed using EndNote 21, and duplicate records were removed to ensure the rigor and reproducibility of the screening process. Following the predefined inclusion and exclusion criteria, two researchers independently conducted the initial screening of studies by reviewing titles and abstracts. Full texts of potentially eligible studies were retrieved for further assessment. Prior to final screening, the two researchers calibrated their understanding using the PICOS (Population, Intervention, Comparison, Outcome, Study Design) framework and refined decision rules to ensure methodological consistency in study inclusion. Any disagreements between the researchers were resolved through discussion with a third researcher.

Two researchers independently extracted the following data: (1) basic information, including first author, publication year, and country; (2) participant characteristics, including sample size, age, and weight; (3) intervention details: name of hemostatic regimen, dosage, and timing of administration; (4) outcomes: 24-hour blood loss, requirements for RBC, PLT, and FFP transfusion, mortality, re-sternotomy for hemostasis, thrombosis, and renal dysfunction. Any discrepancies in data extraction were resolved by consensus with a third researcher.

### 2.4 Quality assessment

For included RCTs, the risk of bias was assessed using the Cochrane Risk of Bias tool 2.0 (RoB 2.0) (26). This tool evaluates bias across five core domains: (1) selection bias (random sequence generation and allocation concealment); (2) performance bias; (3) detection bias; (4) attrition bias; and (5) reporting bias. For included cohort studies, the risk of bias was assessed using the Newcastle-Ottawa Scale (NOS). This tool evaluates studies based on three core domains: the selection of the exposed and non-exposed cohorts, the comparability of the cohorts, and the assessment of the outcome and adequacy of follow-up.

### 2.5 Statistical method

All statistical analyses were performed using R software (version 4.3.2). Direct and indirect comparisons were conducted using JAGS software (version 4.3.1). The primary outcomes were specified as 24-hour blood loss and transfusion requirements. Secondary outcomes included mortality, re-sternotomy for hemostasis, thrombosis, and renal dysfunction. Pairwise meta-analyses for all comparisons were first conducted, and a NMA based on a Bayesian framework was then performed using Markov chain Monte Carlo (MCMC) methods via the JAGS software (version 4.3.1) and the GeMTC, metafor, and meta packages in R. All models employed a random-effects approach. For continuous outcomes, the standardized mean difference (SMD) with a 95% confidence interval (CI) was used as the effect measure. Both consistency and inconsistency models were fitted for all outcomes. The overall consistency of the network was evaluated by comparing the deviance information criterion (DIC) values between these two models, and the model with the lower DIC value was selected for the final analysis. Local inconsistencies between direct and indirect comparisons were assessed using the node-splitting method (*P* > 0.05 indicates local consistency) (27). The surface under the cumulative ranking curve (SUCRA) was calculated to quantify the probability of each hemostatic strategy being the best treatment option, with SUCRA values ranging from 0% to 100% and positively correlated with the ranking of the interventions.

Network plots were generated using Stata 15.0. In the plots, the width of the connecting lines between different interventions was proportional to the number of included trials, and the size of the nodes was proportional to the total sample size. The absence of a connecting line between two interventions indicated no direct comparison, allowing for indirect comparison within the NMA. Publication bias was assessed using funnel plots.

Evidence quality was assessed using CINeMA (Confidence in Network Meta-Analysis), a framework developed based on the GRADE approach (https://cinema.ispm.unibe.ch). This tool evaluates the certainty of a network meta-analysis across the following six key domains: risk of bias within studies, reporting bias, indirectness, imprecision, heterogeneity, and incoherence. For each domain, the level of concern can be classified as no concern (no downgrading), some concern (downgraded by one level), or major concern (downgraded by two levels). The final certainty rating of the NMA evidence aligns with the GRADE system and is categorized as high, moderate, low, or very low (28).

## 3 Results

### 3.1 Study selection

A total of 8,280 records were identified. After removing 1,542 duplicates and 3,024 records marked as ineligible by automation tools, 3,714 records remained for initial screening. Following title and abstract review, 3,620 records were excluded based on PICO criteria, leaving 94 records for full-text retrieval. Five records were excluded as the full texts could not be obtained. Consequently, 89 full-text articles were assessed for eligibility. Ultimately, 27 studies were included (29–55). See Supplementary Figure S1.

### 3.2 Study and patient characteristics

A total of 27 studies involving 4,556 patients were included. Fourteen studies were RCTs (32–36, 38, 40, 44–46, 48, 51, 52, 55), and the remaining thirteen were cohort studies (29–31, 37, 39, 41–43, 47, 49, 50, 53, 54). Ten studies exclusively enrolled neonates (31, 34, 37, 38, 41–43, 47, 55, 56), while the other seventeen studies included infants (29, 30, 32, 35, 36, 40, 44–46, 48–54, 57).

Regarding the hemostatic interventions, the treatment arms in the included studies employed: TXA in one study (41); EACA in two studies (42, 43); APR in ten studies (29, 30, 32, 38, 39, 41, 45, 46, 50, 55); FFP in five studies (40, 44, 48, 53, 54); FC in six studies (33, 34, 36, 49, 51, 52); PCC in one study (37); rFVIIa in two studies (31, 35); and a4FPCC in one study (47). See Table 1.

**Table 1:** Basic characteristics of included studies.

Author	Year of Publication	Year of patient enrollment	Country	Study type	N (T/C)	Age（T/C）	Weight (T/C) kg	Intervention	Cumulative dose	Timing of medication	Control	Outcome
Bojan (29)	2012	2007-2010	France	Retrospective cohort study	390/568	T:96.59 ± 61.74/d C:103.16 ± 57.97/d	T:4.4 ± 1.5 C:4.5 ± 1.5	APR	Loading dose: 30000 KIU/kg; Pre filled solution: 30000 KIU/kg; Continuous infusion: 8000 KIU/kg/h	Perioperative	Without Aprotinin	Mortality, re-sternotomy for hemostasis, renal dysfunction
Breuer (30)	2009	2005-2006	Germany	Prospective Cohort Study	85/114	T:260.07 ± 378.58/d C:250.26 ± 412.21/d	T:6.05 ± 3.24 C:6.34 ± 4.88	APR	Loading dose: 50000 KIU/kg; Pre filling solution: 100000 KIU/100 ml； Continuous infusion : 10000 KIU/kg/h	Perioperative	TXA	24-hour bleeding loss, transfusion requirements (RBC, PLT, and FFP), mortality, re-sternotomy for hemostasis
Christoff (31)	2019	2007-2015	Australia	Retrospective study	129/287	T:5.35 ± 5.25/d C:7.70 ± 5.96/d	T:3.16 ± 0.52 C:3.3 ± 0.44	frFVIIa	100μg/kg	Postoperative	Conventional treatment	Mortality, thrombosis
Davies (32)	1997	NA	Britain	RCT	4/6	T:22 ± 24/w C:39 ± 13/w	T:5.9 ± 2.3 C:6.7 ± 1.8	APR	Load dose o: 140 KIU/m ²; Pre filled solution of 240 KIU/m ²: Continuous infusion of 56 KIU · m ⁻² · h ⁻¹	Perioperative	Placebo	24-hour bleeding loss, transfusion requirements (RBC)
Downey (33)	2020	2016-2018	America	RCT	30/29	T:4.36 ± 3.89/m C:3.64 ± 2.34/m	T:6.16 ± 1.63 C:5.19 ± 1.48	FC	107.8 mg/kg	Postoperative	Cryoprecipitate	24-hour bleeding loss, transfusion requirements (RBC, PLT, and FFP), mortality, re-sternotomy for hemostasis, thrombosis
Downey (34)	2025	NA	America	RCT	18/18	T:5.36 ± 2.41/d C:8.09 ± 6.27/d	T:3.11 ± 0.74 C:3.36 ± 0.62	FC	96 mg/kg	Postoperative	Cryoprecipitate	Transfusion requirements (RBC, PLT, and FFP), mortality, re-sternotomy for hemostasis, thrombosis
Ekert (35)	2006	NA	Australia	RCT	40/35	T:4.0/m C:3.9/m	T:5.2 C:5.3	factor VII	40μg/kg	Postoperative	Placebo	Transfusion requirements (RBC, PLT, and FFP), renal dysfunction
Fiedorek (36)	2020	NA	America	RCT	18/15	T:3.05 ± 3.33/m C:2.39 ± 3.01/m	T:5.34 ± 2.05 C:4.33 ± 1.88	FC	105.76μg/kg	-	Cryoprecipitate	24-hour bleeding loss, transfusion requirements (PLT)
Giorni (37)	2014	2010-2012	Italy	Prospective Cohort Study	14/11	T:30.83 ± 48.60/d C:18.47 ± 10.18/d	T:3.38 ± 0.91 C:3.49 ± 0.85	PPC	0.1 mg/kg	30 minutes after CPB goes offline	Conventional treatment	24-hour bleeding loss, transfusion requirements (RBC and FFP)
Graham (38)	2012	2007-2009	America	RCT	34/42	T:8.5 ± 1.0/d C:8.9 ± 0.9/d	T:3.2 ± 0.1 C:3.2 ± 0.1	APR	Load dose: 200 mg/m ² Maintenance dose: 50 mg · m ⁻² · h ⁻¹	Perioperative	TXA	transfusion requirements (RBC, PLT, and FFP), renal dysfunction
Guzzetta (39, 56)	2009	2005-2008	America	Retrospective cohort study	156/44	T:6.4 ± 5.1/d C:7.2 ± 5.6/d	T:3.2 ± 0.5 C:3.2 ± 0.6	APR	Load dose: 240 mg/m ²; Pre filled liquid loading dose: 240 mg/m ²; Continuous infusion: 56 mg/m ²/h	Perioperative	Without APR	Mortality, thrombosis, renal dysfunction
Lee (40)	2013	NA	Korea	RCT	26/28	T:4.21 ± 3.69/m C:2.6 ± 1.72/m	T:5.81 ± 2.27 C:5.02 ± 1.64	FFP	1-2 units	Intraoperative	20% albumin	24-hour bleeding loss, transfusion requirements (RBC, PLT, and FFP)
Lin (41)	2015	2003-2008	America	Retrospective study	T1:100 T2:275 C:184	T1:7.41 ± 6.01/d T2:5.70 ± 2.98/d C:7.40 ± 5.98/d	T1:3.26 ± 0.56 T2:3.10 ± 0.66 C:3.06 ± 0.61	T1: TXA T2: APR	T1: Load dose: 100 mg/kg Maintain infusion: 10 mg/kg/h Pre filling solution addition: 100 mg/kg T2: Load dose: 30000 KIU/kg Maintain infusion: 10000 KIU/kg/h Pre filling solution addition: 30000 KIU/kg	Perioperative	Conventional treatment	Mortality, re-sternotomy for hemostasis, renal dysfunction
Martin (42, 43)	2011	NA	Germany	Retrospective study	77/28	T:12 ± 6/d C:11 ± 6/d	T:3.2 ± 0.5 C:3.2 ± 0.5	EACA	Load dose: 75 mg/kg; Pre filling solution addition: 75 mg/100 mL	Perioperative	TXA	24-hour bleeding loss, re-sternotomy for hemostasis
Martin (42, 43)	2011	2006-2009	Germany	Retrospective study	140/95	T:11.3 ± 5.9/d C:11.5 ± 6.6/d	T:3.2 ± 0.5 C:3.3 ± 0.5	EACA	Load dose: 75 mg/kg ; Pre filling solution addition: 75 mg/100 mL	Perioperative	APR	24-hour bleeding loss, transfusion requirements (RBC and FFP), mortality, re-sternotomy for hemostasis, thrombosis, renal dysfunction
McCall (44)	2004	NA	America	RCT	10/10	T:4.0 ± 3.9/m C:4.4 ± 1.2/m	T:4.0 ± 1.3 C:4.6 ± 0.9	FFP	Pre filling solution addition: Add 1 unit of FFP (average volume 252 ± 46 mL); Albumin addition: 25% albumin 40 ± 29 mL	Perioperative	25% albumin	24-hour bleeding loss, transfusion requirements (RBC, PLT, and FFP)
Mo ¨ssinger (45)	2003	NA	Germany	RCT	30/30	T:163/d C:123/d	T:5.0 C:4.8	APR	Intravenous injection after aortic catheterization:3 × 10 4kIU/kg； Pre filled liquid loading dose:5 × 10 4kIU/kg	Perioperative	Placebo	Mortality
Murugesan (46)	2008	2006-2007	India	RCT	25/25	T:40 ± 1.8/d C:38 ± 1.5/d	T:3.2 ± 1.2 C:3.2 ± 1.4	APR	40,000 KIU/kg	Postoperative	Placebo	24-hour bleeding loss, transfusion requirements (RBC, PLT, and FFP), mortality, re-sternotomy for hemostasis
Navaratnam (47)	2023	2014-2018	America	Retrospective cohort study	43/43	T:6.7 ± 6.3/d C:5.7 ± 4.5/d	T:3.3 ± 3.2 C:3.16 ± 2.07	a4FPCC	10 IU/kg each time (adjusted to start at 5 IU/kg later in the study)	Postoperative	Conventional treatment	24-hour bleeding loss, mortality, thrombosis, renal dysfunction
Oliver (48)	2003	NA	America	RCT	28/28	T:6.9 ± 7.4/m C:6.6 ± 5.9/m	T:5.6 ± 2.0 C:5.6 ± 1.6	FFP	1UI	Perioperative	5% albumin	24-hour bleeding loss, transfusion requirements (RBC, PLT, and FFP), mortality, re-sternotomy for hemostasis
Rizza (49)	2025	2020-2022	Italy	Retrospective cohort study	70/69	T:6.1 ± 7.1/m C:6.6 ± 7.2/m	T:5.26 ± 2.48 C:5.98 ± 2.64	FC	30 mg/kg	Postoperative	Without FC	24-hour bleeding loss, transfusion requirements (RBC, PLT, and FFP), mortality, re-sternotomy for hemostasis
Scott (50)	2014	2006	America	Retrospective cohort study	68/77	T:2.86 ± 3.86/m C:2.52 ± 3.85/m	T:4.65 ± 1.89 C:4.39 ± 2.04	APR	Load dose: 1.7 × 10 ⁶ KIU/m ² IV; CPB pre charge: 1.7 × 10 ⁶ KIU/m ²; Continuous infusion: 4 × 10 ⁶ KIU/m ²/hr	Perioperative	EACA	24-hour bleeding loss, mortality, re-sternotomy for hemostasis, thrombosis, renal dysfunction
Siemens (51)	2020	NA	Britain	RCT	60/30	T:6.0 ± 5.1/m C:6.9 ± 7.2/m	T:5.9 ± 2.1 C:5.8 ± 2.2	FC	114 mg/kg	Postoperative	Placebo	24-hour bleeding loss, re-sternotomy for hemostasis, thrombosis
Tirotta (52)	2020	2017-2018	America	RCT	15/15	T:148 ± 70.25/d C:146 ± 80.75/d	T:5.3 ± 1.03 C:5.4 ± 1.35	FC	70 mg/kg	Postoperative	Placebo	24-hour bleeding loss, transfusion requirements (RBC, PLT, and FFP), thrombosis
van Minnen (53)	2024	2019-2021	the Netherlands	Retrospective cohort study	90/53	T:67.95 ± 105.48/d C:213.40 ± 136.40/d	T:4.66 ± 1.95 C:6.54 ± 1.88	FFP	45 mL	Postoperative	Omniplasma	24-hour bleeding loss, mortality, thrombosis
Wang (54)	2018	2012-2013	China	Retrospective cohort study	336/335	T:6.30 ± 2.98/m C:7 ± 2.98/m	T:6.55 ± 1.27 C:6.60 ± 1.56	FFP	100-200ml	Postoperative	Gelofusine	24-hour bleeding loss, transfusion requirements (RBC and FFP), mortality
Williams (55)	2008	NA	America	RCT	13/13	T:13 ± 8/d C:6 ± 4/d	T:3.11 ± 0.46 C:3.42 ± 0.56	APR	Load dose: 1.7 × 10 ⁶ kIU/m ² (240 mg/m ²); Add the same dosage to the CPB pre filling solution; Continuous infusion: 4 × 10 ⁵ kIU · m ⁻² · h ⁻¹ (56 mg · m ⁻² · h ⁻¹)	Perioperative	Placebo	24-hour bleeding loss, transfusion requirements (RBC, PLT, and FFP), mortality, thrombosis

a4FPCC, 4-factor prothrombin complex concentrate; APR, aprotinin; C, control group; EACA, ε-aminocaproic acid; FFP, fresh frozen plasma; FC, fibrinogen concentrate; PLT, Platelet; rFVIIa, factor VII; RBC, red blood cell; RCT, randomized controlled trial; T, intervention group; TXA, Tranexamic acid.

### 3.3 Quality assessment

Of the 14 included RCTs, the overall risk of bias was assessed as low in 6 studies, moderate in 6, and high in 2 based on the RoB 2.0 tool (Supplementary Figure S2). For the 13 included cohort studies, the methodological quality was rated as high in 8 studies and moderate in 5 based on the NOS (Supplementary Table S3). The domain-level judgments and overall certainty ratings at the study level are presented in Supplementary Table S4. For outcomes including transfusion requirements (RBC, PLT, FFP), re-sternotomy for hemostasis, thrombosis, and renal dysfunction, during the sensitivity analysis stage, the number of studies meeting the “low risk of bias” criteria was insufficient (fewer than three). As a result, the model failed to converge (the Fisher scoring algorithm did not converge), and therefore subgroup analyses restricted to low-risk studies could not be performed.

### 3.4 24-hour bleeding loss

**Figure 1:**
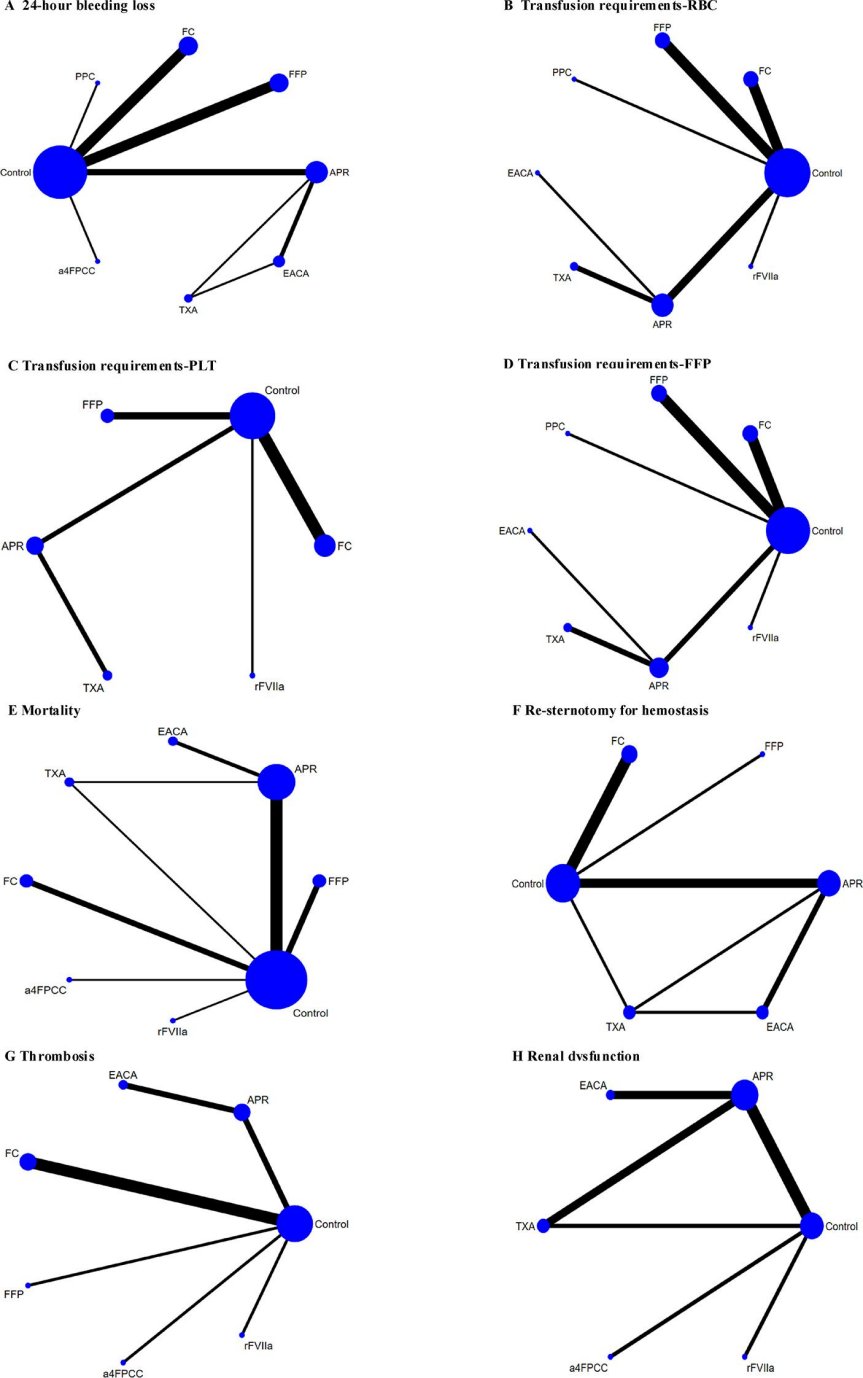
Network plots based on different hemostasis strategy. **(A)** 24-hour bleeding loss. **(B)** Transfusion requirements-RBC. **(C)** Transfusion requirements-PLT. **(D)** Transfusion requirements-FFP. **(E)** Mortality **(F)** Re-sternotomy for hemostasis. **(G)** Thrombosis **(H)** Renal dysfunction.

**Figure 2:**
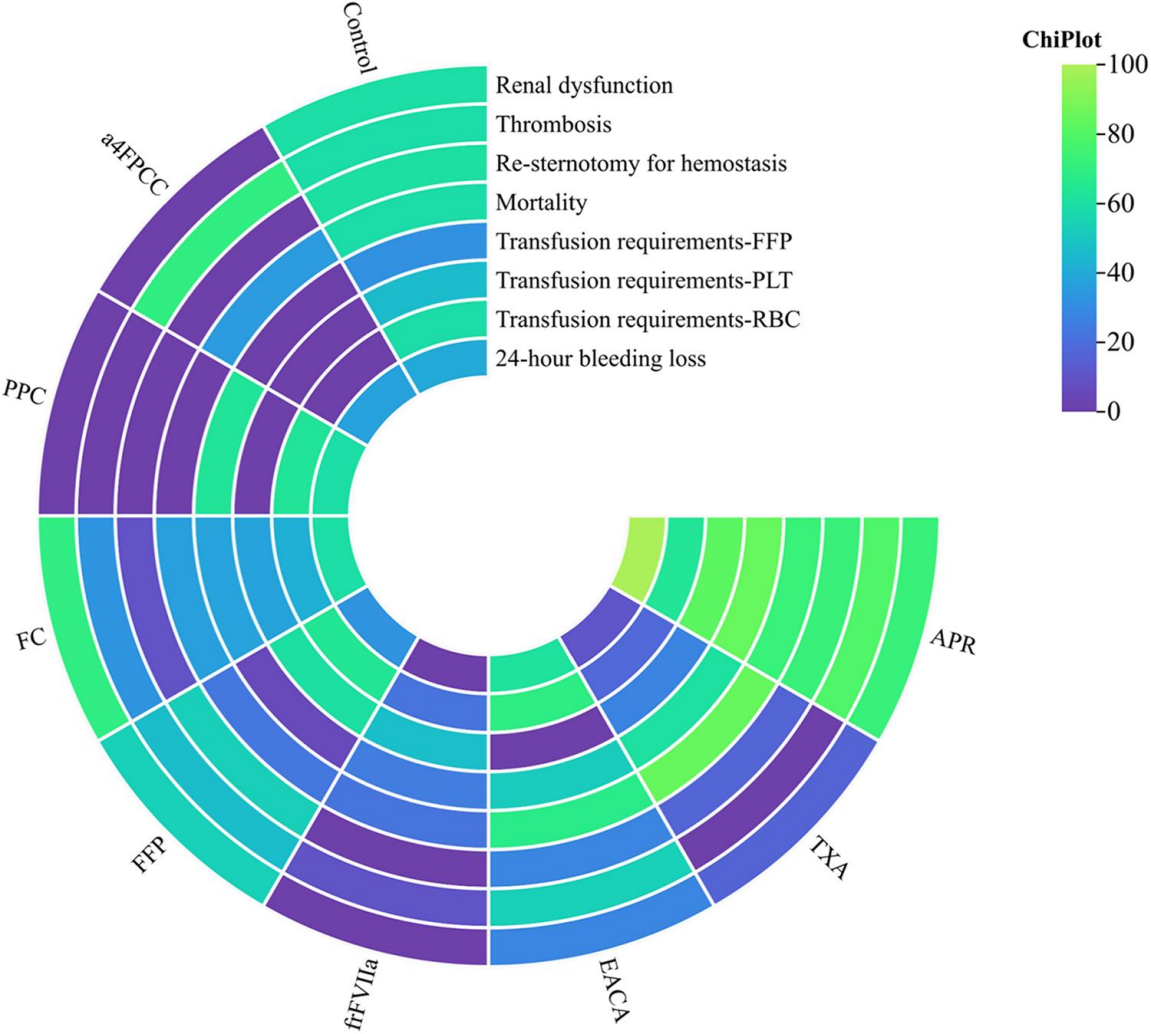
SUCRA results based on heat map.

Data on 24-hour blood loss, one of the primary outcomes, were reported in 19 studies. Among the direct comparisons, FC versus control and FFP versus control were the most frequent, with 5 studies each (Figure 1A). The difference in the DIC between the consistency model (71.43) and the inconsistency model (71.18) was 0.25, indicating good model fit. Results from the pairwise meta-analysis showed that APR demonstrated statistically significant reductions in 24-hour blood loss compared to control [SMD = −12.3, 95% confidence interval (CI): −18.71 to −5.89], FFP (SMD = −12.9, 95% CI: −20.65 to −6.15), FC (SMD = −10.63, 95% CI: −18.42 to −3.14), PCC (SMD = −10.7, 95% CI: −19.29 to −1.99), TXA (SMD = −18.0, 95% CI: −24.78 to −10.63) and EACA (SMD = −9.77, 95% CI: −15.54 to −3.86). Additionally, EACA was significantly superior to TXA in reducing blood loss (SMD = −8.23, 95% CI: −15.91 to −0.37) (Table 2). The NMA ranking indicated that for reducing 24-hour blood loss, APR had the highest SUCRA value (99.15%), making it the optimal intervention, followed by EACA (SUCRA = 70.72%) and FC (SUCRA = 70.72%) (Figure 2).

**Table 2:** Comparison of different hemostatic strategies in reducing 24-hour blood loss

a4FPCC							
13.72 (−1.35, 28.29)	**APR**						
1.47 (−12.36, 14.75)	−12.3 (−18.71, −5.89)*	**Control**					
3.93 (−12.28, 19.74)	−9.77 (−15.54, −3.86)*	2.48 (−6.03, 11.46)	**EACA**				
3.14 (−11.44, 16.92)	−10.63 (−18.42, −3.14)*	1.66 (−2.66, 5.7)	−0.82 (−10.82, 8.46)	**FC**			
0.8 (−13.56, 14.26)	−12.9 (−20.65, −6.15)*	−0.41 (−4.7, 2.08)	−3.08 (−13.14, 5.63)	−2.24 (−7.92, 2.63)	**FFP**		
3.08 (−11.74, 17.52)	−10.7 (−19.29, −1.99)*	1.59 (−4.41, 7.63)	−0.87 (−11.54, 9.37)	−0.1 (−6.98, 7.4)	1.92 (−3.86, 9.95)	**PPC**	
−4.25 (−20.88, 12.09)	−18 (−24.78, −10.63)*	−5.73 (−14.97, 4.09)	−8.23 (−15.91, −0.37)*	−7.39 (−17.28, 3.62)	−5.12 (−14.53, 5.86)	−7.35 (−18, 4.2)	**TXA**

*P < 0.05 for comparison between interventions.

### 3.5 Transfusion requirements-RBC

Data on RBC transfusion requirements, one of the primary outcomes, were reported in 16 studies. Among the direct comparisons, FC versus control and FFP versus control were the most frequent, with 4 studies each (Figure 1B). The difference in the DIC between the consistency model (61.55) and the inconsistency model (61.36) was 0.19, indicating good model fit. Results from the pairwise meta-analysis showed no statistically significant differences for any of the comparisons (Supplementary Table S5). The NMA ranking indicated that for reducing RBC transfusion requirements, EACA had the highest SUCRA value (69.04%), identifying it as the optimal intervention, followed by FFP (SUCRA = 64.15%) and APR (SUCRA = 63.26%) (Figure 2).

### 3.6 Transfusion requirements-PLT

Data on PLT transfusion requirements, one of the primary outcomes, were reported in 13 studies. Among the direct comparisons, FC versus control was the most frequent, with 5 studies (Figure 1C). The difference in the DIC between the consistency model (50.72) and the inconsistency model (51.01) was 0.29, indicating acceptable model fit. Results from the pairwise meta-analysis showed no statistically significant differences for any of the comparisons (Supplementary Table S6). The NMA ranking indicated that for reducing PLT transfusion requirements, APR had the highest SUCRA value (82.19%), identifying it as the optimal intervention, followed by FFP (SUCRA = 60.23%) and rFVIIa (SUCRA = 46.86%) (Figure 2).

### 3.7 Transfusion requirements-FFP

Data on FFP transfusion requirements, one of the primary outcomes, were reported in 15 studies. Among the direct comparisons, FC versus control and FFP versus control were the most frequent, with 4 studies each (Figure 1D). The difference in the DIC between the consistency model (61.25) and the inconsistency model (61.09) was 0.16, indicating good model fit. Results from the pairwise meta-analysis showed no statistically significant differences for any of the comparisons (Supplementary Table S7). The NMA ranking indicated that for reducing FFP transfusion requirements, APR had the highest SUCRA value (84.82%), identifying it as the optimal intervention, followed by PCC (SUCRA = 62.92%) and TXA (SUCRA = 60.86%) (Figure 2).

### 3.8 Mortality

Data on mortality, a secondary outcome, were reported in 17 studies. Among the direct comparisons, APR versus control was the most frequent, with 6 studies (Figure 1E). The difference in the DIC between the consistency model (68.51) and the inconsistency model (69.72) was 1.21, indicating good model fit. Results from the pairwise meta-analysis showed no statistically significant differences for any of the comparisons (Supplementary Table S8). The NMA ranking indicated that for reducing mortality, TXA had the highest SUCRA value (84.77%), identifying it as the optimal intervention, followed by EACA (SUCRA = 72.92%) and APR (SUCRA = 67.55%) (Figure 2).

### 3.9 Re-sternotomy for hemostasis

Data on re-sternotomy for hemostasis, a secondary outcome, were reported in 12 studies. Among the direct comparisons, FC versus control was the most frequent, with 4 studies (Figure 1F). The difference in the DIC between the consistency model (45.79) and the inconsistency model (48.62) was 2.83, indicating acceptable model fit. Results from the pairwise meta-analysis showed no statistically significant differences for any of the comparisons (Supplementary Table S9). The NMA ranking indicated that for reducing the need for re-sternotomy for hemostasis, APR had the highest SUCRA value (72.82%), identifying it as the optimal intervention, followed by the control group (SUCRA = 60.21%) and FFP (SUCRA = 53.05%) (Figure 2).

### 3.10 Thrombosis

Data on thrombosis, a secondary outcome, were reported in 11 studies. Among the direct comparisons, FC versus control was the most frequent, with 4 studies (Figure 1G). The difference in the DIC between the consistency model (37.95) and the inconsistency model (38.07) was 0.12, indicating good model fit. Results from the pairwise meta-analysis showed a statistically significant difference for the comparison between control and recombinant activated rFVIIa (RR = 0.27, 95% CI: 0.07 to 0.98), indicating a lower incidence of thrombosis in the control group (Table 3). The NMA ranking indicated that for reducing thrombosis, APR had the highest SUCRA value (79.13%), identifying it as the optimal intervention, followed by a4FPCC (SUCRA = 69.93%) and the control group (SUCRA = 58.5%) (Figure 2).

**Table 3:** Comparison of different hemostatic strategies in thrombosis

a4FPCC						
1.09 (0.07, 13.74)	**APR**					
0.64 (0.06, 5.23)	0.59 (0.14, 2.64)	**Control**				
0.63 (0.03, 10.19)	0.59 (0.17, 1.78)	1.01 (0.14, 5.94)	**EACA**			
0.37 (0.03, 3.76)	0.34 (0.06, 2.08)	0.57 (0.2, 1.57)	0.58 (0.07, 5.37)	**FC**		
0.47 (0.02, 8.99)	0.44 (0.03, 5.67)	0.76 (0.08, 5.7)	0.75 (0.04, 12.97)	1.34 (0.11, 13.09)	**FFP**	
0.17 (0.01, 2.00)	0.16 (0.02, 1.14)	0.27 (0.07, 0.98)*	0.27 (0.03, 2.83)	0.48 (0.09, 2.42)	0.36 (0.03, 4.84)	**rFVIIa**

*P < 0.05 for comparison between interventions.

### 3.11 Renal dysfunction

Data on renal dysfunction, a secondary outcome, were reported in 9 studies. Among the direct comparisons, APR versus control was the most frequent, with 3 studies (Figure 1H). The difference in the DIC between the consistency model (45.83) and the inconsistency model (48.51) was 2.68, indicating acceptable model fit. Results from the pairwise meta-analysis showed a statistically significant difference for the comparison between APR and TXA (RR = 0.40, 95% CI: 0.15 to 0.98), indicating a lower incidence of renal dysfunction with APR (Table 4). The NMA ranking indicated that for reducing renal dysfunction, APR had the highest SUCRA value (72.61%), identifying it as the optimal intervention, followed by FC (SUCRA = 69.80%) and the control group (SUCRA = 59.83%) (Figure 2).

**Table 4:** Comparison of different hemostatic strategies in renal dysfunction

APR					
0.86 (0.4, 1.64)	**Control**				
0.52 (0.23, 1.04)	0.59 (0.23, 1.61)	**EACA**			
1.07 (0.28, 4.18)	1.24 (0.4, 4.19)	2.11 (0.48, 9.91)	**FC**		
0.84 (0.02, 39.6)	0.98 (0.02, 41.78)	1.65 (0.03, 79.74)	0.78 (0.01, 40.52)	**FFP**	
0.40 (0.15, 0.99)*	0.47 (0.17, 1.29)	0.78 (0.29, 2.1)	0.37 (0.08, 1.72)	0.47 (0.01, 24.61)	**TXA**

*P < 0.05 for comparison between interventions.

### 3.12 Node-splitting analysis

Node-splitting analyses for outcomes including 24-hour blood loss, mortality, re-sternotomy for hemostasis, and renal dysfunction revealed no local inconsistency for any outcome (*P* > 0.05), indicating high agreement between direct and indirect evidence estimates. See Supplementary Figure S3.

### 3.13 Publication bias

Publication bias was assessed using funnel plots for outcomes including 24-hour blood loss, transfusion requirements for RBC, PLT, and FFP, mortality, re-sternotomy for hemostasis, thrombosis, and renal dysfunction. All funnel plots exhibited symmetrical shapes, suggesting no significant publication bias. See Supplementary Figure S4.

### 3.14 Sensitivity analysis

We excluded cohort studies and retained RCTs for sensitivity analysis to assess the robustness of the results. The findings demonstrated that after the sequential exclusion of each study, the outcomes for 24-hour bleeding loss, transfusion requirements (RBC, PLT, and FFP), mortality, re-sternotomy for hemostasis, and thrombosis remained stable. For the outcome of renal dysfunction, sensitivity analysis was not performed because it was reported in only one RCT. See Supplementary Figures S1–S7.

## 4 Discussion

This study represented the first NMA to comprehensively evaluate the effects of different hemostatic strategies in neonates and infants undergoing cardiac surgery with CPB, integrating evidence from both RCTs and cohort studies across three key domains: hemostatic efficacy, patient prognosis, and safety. The findings demonstrated that APR was the optimal hemostatic strategy for reducing 24-hour blood loss, PLT and FFP transfusion requirements, re-sternotomy for hemostasis, thrombosis, and renal dysfunction. EACA was identified as the best strategy for reducing RBC transfusion needs, while TXA emerged as the most effective intervention for reducing mortality.

Overall, antifibrinolytic agents showed potential benefits in hemostasis, prognosis, and safety for this population. Current guidelines for blood management in cardiac surgery primarily focus on adult populations (58, 59), with limited attention to neonates and infants. Therefore, this study aimed to fill this critical evidence gap and provide valuable guidance for optimizing clinical outcomes in this vulnerable group.

Our findings differ from and extend previous systematic reviews and meta-analyses in the following aspects (9, 21, 23). First, while Schertz et al. and Siemens et al. included children up to 18 years of age, our review specifically focused on neonates and infants with a mean age ≤12 months. This narrower age range reduces age-related heterogeneity and provides evidence that is more directly applicable to the youngest and most vulnerable population undergoing cardiac surgery with cardiopulmonary bypass. Second, our review included a broader range of interventions, not limited to antifibrinolytic agents but also including plasma products (FFP, FC, PPC) and other hemostatic agents, allowing for a more comprehensive comparison of strategies for bleeding prevention in this specific age group. Third, unlike Zou et al., who specifically examined the effect of TXA on platelet counts and function in a mixed adult-pediatric population, our review focuses on clinical outcomes (bleeding and transfusion) in a purely neonatal/infant population. While Zou et al. provided mechanistic insights showing that TXA improved ADP-stimulated platelet aggregation and CD63 expression in pediatric patients undergoing cardiac surgery with cardiopulmonary bypass, our study complements these findings by demonstrating the corresponding clinical benefits in terms of reduced blood loss and transfusion requirements. In summary, in terms of main findings and results, our review does not contradict previous evidence but rather refines and complements it by focusing on the highest-risk age subgroup (neonates and infants) and by including a broader range of hemostatic interventions.

Our findings demonstrated that APR was the optimal hemostatic strategy in terms of efficacy and safety, which was consistent with the results reported by Atasever et al. (22). Their study found that APR reduced blood loss, transfusion requirements, and the need for resternotomy in cardiac surgery, while also lowering the risk of myocardial infarction. Patients undergoing cardiac surgery with CPB experience increased fibrin breakdown, leading to an imbalance between fibrin formation and degradation and resulting in a hyperfibrinolytic state (60). Considering the differences between pediatric and adult hemostatic systems (23), this situation may be even more critical. As a primary antifibrinolytic agent, APR significantly inhibits the generation and activity of plasmin through its antiplasmin and antikallikrein effects, thereby reducing blood loss, transfusion needs, and the necessity for reoperation (61). Furthermore, CPB in neonates and infants leads to more severe hemodilution, greater blood exposure to artificial surfaces, higher shear stress (due to higher CPB flow rates), and longer bypass and aortic cross-clamp times, which collectively promote platelet activation, coagulation, and a decline in both platelet count and function (62).

Our study found that APR significantly reduced the requirements for PLT and FFP transfusions in neonates and infants. This may be attributed to APR's platelet-protective effect, which mitigates CPB-induced platelet dysfunction, thereby reducing PLT demand and indirectly decreasing the need for FFP transfusion. According to Vanglabeke et al. (63), this platelet-protective effect of APR could be due to multiple mechanisms, including the inhibition of platelet aggregation [via protection of glycoprotein (GP) IIb/IIIa receptors], inhibition of platelet adhesion (by protection of GP Ib receptors), protection of both aggregation and adhesion receptors, proteolysis of protease-activated receptor 1, and inhibition of platelet activation through the suppression of plasmin and thrombin.

In summary, APR exerts its significant clinical effects—including reducing postoperative bleeding, PLT and FFP transfusion requirements, and the risk of reoperation—through a synergistic triple mechanism of antifibrinolysis, anti-inflammation, and platelet protection. However, it is important to note that APR is currently not approved for pediatric use in many regions (e.g., the USA), and its use in Europe remains restricted to high-risk adult CABG patients only. Therefore, while APR demonstrates promising efficacy in neonates and infants undergoing complex cardiac surgery, its clinical applicability is limited by regulatory restrictions. Any off-label use in pediatric patients should be guided by local regulations, institutional protocols, and a careful individual risk-benefit assessment.

Controversy still exists surrounding the benefits and risks of APR, and evidence has not been consolidated to validate its effects specifically in children under one year of age undergoing cardiac surgery with CPB. Our study found that APR significantly reduced the risk of renal dysfunction compared to TXA (*P* < 0.05). For reducing thrombosis, no statistically significant differences were observed between APR and any comparator; the SUCRA ranking indicated that APR was the optimal intervention. Similarly, Arnold et al. confirmed no correlation between APR therapy and thrombosis in a meta-analysis of 12 studies involving pediatric patients undergoing CPB cardiac surgery; among the seven studies that reported adverse events, no thrombosis was observed to be induced by APR treatment (64). This may have been related to APR's effect of reducing postoperative blood loss and transfusion requirements, thereby diminishing the conditions conducive to thrombosis. Bojan et al. found that after adjusting for the use of miniaturized circuits and year of surgery, APR was not significantly associated with the incidence of acute kidney injury (AKI) in neonates and infants undergoing cardiac surgery with CPB (29). Guzzetta et al. found no significant difference in the incidence of renal dysfunction between those who received APR and those who did not (39). Further multivariate analysis identified a CPB duration greater than 100 min as a key predictor of postoperative renal dysfunction, rather than APR administration (39). In summary, our findings contrasted with previous concerns observed in adult patients that APR treatment might increase the risks of thrombosis and renal dysfunction (65). This discrepancy may be attributed to important pathophysiological differences between age groups in coagulation, inflammatory responses, and drug reactions.

Our findings indicated that EACA was the optimal hemostatic strategy for reducing RBC transfusion requirements. Siemens et al. and Schertz et al., in separate meta-analyses each including three RCTs, reported that EACA significantly decreased RBC transfusion needs in pediatric patients undergoing cardiac surgery with CPB, with an average reduction of 11.3 mL/kg/24 h (9, 23). By inhibiting fibrinolysis, EACA helped maintain the stability of intraoperative clot formation, thereby achieving the goals of reducing blood loss and transfusion requirements (66). However, this finding required cautious interpretation. Our analysis included only a single study on this specific comparison, which may have resulted in an unstable effect estimate. Therefore, future high-quality, large-scale studies remain necessary to validate this finding and provide more robust evidence to guide clinical decision-making.

Our study found that TXA was the optimal intervention for reducing mortality. Multiple studies had demonstrated that TXA did not increase the risk of patient mortality while effectively reducing blood loss and transfusion requirements in cardiac surgery (67, 68). Dumas et al. evaluated the effect of TXA in trauma patients at risk of bleeding in both pre-hospital and in-hospital settings. They found that TXA significantly reduced 24-hour mortality in both environments (69). TXA has been widely used for perioperative hemostasis in cardiac surgery. However, existing research on TXA in pediatric cardiac surgery has primarily focused on its effects on outcomes such as blood loss and transfusion requirements, with mortality rarely reported as a primary outcome. This may be attributed to the need for larger sample sizes and long-term follow-up. Consequently, our finding still requires future validation through large-scale RCTs to confirm its impact on the long-term survival of pediatric patients.

### 4.1 Limitations

This study had several limitations. First, heterogeneity might have influenced the results due to variations in CPB protocols, underlying diseases, surgical types, preexisting bleeding tendencies, dosages of hemostatic strategies, and timing of administration. Second, safety outcomes such as seizures, stroke, and allergic reactions could not be assessed due to insufficient reporting in the included studies. Third, some RCTs were methodologically limited due to the lack of explicit blinding and follow-up data. Fourth, the reporting units for outcomes such as 24-hour blood loss and transfusion requirements were inconsistent, precluding a precise quantitative estimation of effect sizes. Fifth, the limited sample sizes in some studies might have affected the reliability of the findings. Sixth, considering that the significant treatment effect of APR might be influenced by era effects, we attempted to perform a traditional meta-regression to evaluate the impact of enrollment year on the treatment effect of APR. However, the number of included studies for the primary outcomes was fewer than 10. Under these circumstances, a meta-regression is statistically underpowered and may produce unstable and unreliable estimates. Therefore, future head-to-head randomized controlled trials directly comparing APR with FC or FFP are urgently needed to validate our findings. Seventh, for the outcome of renal dysfunction, the included studies used different diagnostic criteria to define renal dysfunction, including KDIGO, pRIFLE, and AKIN criteria. The lack of a harmonized definition across studies may have introduced clinical heterogeneity and could potentially bias the pooled effect estimate. Therefore, the results for this outcome should be interpreted with caution. Future studies should adopt a uniform, widely accepted definition of renal dysfunction to facilitate meaningful comparisons and meta-analysis. Eighth, some of the included studies, such as Davies 1997, had very small sample sizes (e.g., 4 patients in the intervention group and 6 in the control group). This may result in limited statistical precision and increased susceptibility to random error. Future large-scale, well-designed studies are needed to confirm these findings. Finally, recently published studies have predominantly focused on blood component therapies such as FC and FFP, which may have introduced an era bias, as earlier studies investigating antifibrinolytic agents might have employed surgical and perioperative management practices that differ from current standards.

## 5 Conclusion

Antifibrinolytic agents may demonstrated significant hemostatic efficacy and a generally acceptable safety profile in neonates and infants undergoing cardiac surgery with CPB. APR showed promising efficacy in reducing bleeding and transfusion requirements. However, due to regulatory restrictions (not approved for pediatric use in many regions), evidence heterogeneity, and the partially observational nature of the included studies, APR may be considered as a potential option, but its use requires caution and strict adherence to local regulations and careful risk-benefit assessment. TXA, which is widely available and licensed for pediatric use, remains a practical first-line choice. In the available network, blood component therapies did not emerge as consistently superior strategies compared with other hemostatic interventions. For 24-hour blood loss, APR was associated with lower blood loss than FFP, FC, and PCC. However, because the relevant comparisons were largely against active comparators rather than placebo or no hemostatic intervention, the efficacy of blood component therapies relative to no blood component therapy remains uncertain. Future large-scale RCTs are needed to further validate these findings.
